# MAPK15 Controls Hedgehog Signaling in Medulloblastoma Cells by Regulating Primary Ciliogenesis

**DOI:** 10.3390/cancers13194903

**Published:** 2021-09-29

**Authors:** Silvia Pietrobono, Lorenzo Franci, Francesco Imperatore, Cristina Zanini, Barbara Stecca, Mario Chiariello

**Affiliations:** 1 Core Research Laboratory—Firenze, Institute for Cancer Research and Prevention (ISPRO), 50139 Firenze, Italy; silvia.pietrobono@ittumori.it; 2 Core Research Laboratory—Siena, Institute for Cancer Research and Prevention (ISPRO), 53100 Siena, Italy; franci36@student.unisi.it (L.F.); imperatore@ifc.cnr.it (F.I.); 3 Institute of Clinical Physiology (IFC), National Research Council (CNR), 53100 Siena, Italy; 4 Department of Medical Biotechnologies, University of Siena, 53100 Siena, Italy; 5 Molecular Biotechnology Center, BioAir S.p.A Research Laboratory, University of Turin, 10126 Torino, Italy; c.zanini@bioair.it

**Keywords:** MAP Kinases, primary cilia, cancer stem cells, medullo-spheres, GLI1

## Abstract

**Simple Summary:**

In eukaryotes, MAPK15 controls the assembly of primary cilia, which are microtubule-based cell surface organelles necessary for sensing and processing developmental signals as well as for transducing tumorigenic Hedgehog signaling in medulloblastomas and basal cell carcinomas. The aim of this study was to evaluate the role of MAPK15 in regulating Hedgehog signaling in medulloblastoma cells. Indeed, we demonstrated strict dependency on this kinase of medulloblastoma ciliogenesis and Hedgehog signaling, which resulted in a reduced cancer stem cell compartment. Based on the scarce therapeutic options available for medulloblastoma patients, our data support the possibility of exploiting novel pharmacological approaches targeting this often-underestimated MAP kinase.

**Abstract:**

In medulloblastomas, genetic alterations resulting in over-activation and/or deregulation of proteins involved in Hedgehog (HH) signaling lead to cellular transformation, which can be prevented by inhibition of primary ciliogenesis. Here, we investigated the role of MAPK15 in HH signaling and, in turn, in HH-mediated cellular transformation. We first demonstrated, in NIH3T3 mouse fibroblasts, the ability of this kinase of controlling primary ciliogenesis and canonical HH signaling. Next, we took advantage of transformed human medulloblastoma cells belonging to the SHH-driven subtype, i.e., DAOY and ONS-76 cells, to ascertain the role for MAPK15 in HH-mediated cellular transformation. Specifically, medullo-spheres derived from these cells, an established in vitro model for evaluating progression and malignancy of putative tumor-initiating medulloblastoma cells, were used to demonstrate that MAPK15 regulates self-renewal of these cancer stem cell-like cells. Interestingly, by using the HH-related oncogenes SMO-M2 and GLI2-DN, we provided evidences that disruption of MAPK15 signaling inhibits oncogenic HH overactivation in a specific cilia-dependent fashion. Ultimately, we show that pharmacological inhibition of MAPK15 prevents cell proliferation of SHH-driven medulloblastoma cells, overall suggesting that oncogenic HH signaling can be counteracted by targeting the ciliary gene MAPK15, which could therefore be considered a promising target for innovative “smart” therapies in medulloblastomas.

## 1. Introduction

Medulloblastoma (MB) is an embryonal neuroepithelial tumor of the cerebellum and the most common pediatric malignant brain tumor. Four main molecular subgroups have been described: WNT (Wingless)-driven, SHH-driven, G3 (Group 3), and G4 (Group 4) MBs [1,2,3]. Therapeutic approaches for MB consist mainly of surgical resection; chemotherapy; and, for patients over the age of 3, craniospinal irradiation. These methods have improved survival; however, they are frequently associated with severe long-term adverse effects, and approximately one-third of patients still die from the disease [4]. Therefore, there is an urgent need to understand the mechanisms sustaining MB growth and maintenance to devise novel and efficacious therapeutic approaches for this disease.

MAPK15 (ERK7; ERK8) is still a poorly characterized protein of the mitogen activated protein (MAP) kinase family localized to several intracellular compartments [5,6], among which are the basal bodies of motile and primary cilia [7,8]. In the different locations, MAPK15 controls several important cellular functions, which are often related to the management of cell stress and to cancer development. Specifically, this MAP kinase has been involved in the control of DNA damage responses [9,10], autophagy, and protein secretion [6,11], and correlated to transformation in colon [12] and testis [10] tumors as well as in chronic myeloid leukemia [13]. Importantly, MAPK15 was recently involved in ciliogenesis, a multistep process culminating in the assembly of microtubule-based organelles on the cell surface, which is important for sensing and processing developmental signals (the primary cilia) or directing the flow of fluids in major organs of mammals (the multi-cilia of specialized epithelial cells) [14].

The primary cilium has been repeatedly demonstrated to control a number of “core” signaling pathways, among which are those initiated by Sonic hedgehog (SHH), platelet-derived growth factor (PDGF)-AA, WNT, and NOTCH [15]. The best-studied cilia-linked pathway is the Hedgehog (HH) signaling, which plays fundamental roles in growth and tissue patterning during embryonic development [16,17], while during adult life it is mostly involved in regulating tissue homeostasis and stem cell behavior [18,19]. Indeed, several proteins participating in the initial intracellular signaling induced by SHH are strongly compartmentalized into the primary cilium, and mutations in different ciliary proteins perturb SHH signaling [15].

Canonical HH signaling is initiated by the binding of the HH ligands (Sonic hedgehog, Indian hedgehog, and Desert hedgehog) to the 12 transmembrane protein Patched1 (PTCH1), which is localized on the primary cilium. Upon ligand binding, PTCH1 exits the primary cilium and releases the inhibition on the main Hedgehog pathway transducer, the G protein-coupled receptor Smoothened (SMO), allowing its translocation into the primary cilium. At this point, active SMO triggers an intracellular signaling cascade promoting the functions of the zinc finger transcription factors GLI2 and GLI3. Dissociation of these transcription factors from SUFU leads to their full activation and translocation into the nucleus, where they begin transcription of HH pathway target genes, including GLI1 [20]. Interestingly, human MBs and basal cell carcinomas (BCCs) are frequently ciliated and abnormal activation of canonical HH signaling, through loss of the HH receptor PTCH1 or activation of SMO, is frequently found in human patients and is sufficient to induce these tumors in mice [21,22]. More importantly, clear demonstrations that ablation of primary cilia blocks the development of MBs and BCCs, when induced by these mutations, have been obtained [21,22], suggesting that targeting the machinery controlling the assembly of primary cilia may represent a potentially successful strategy to restrain tumors with specific genetic alterations, particularly among MBs and BCCs.

Here, we demonstrate that HH signaling is strongly dependent on MAPK15 ability to control primary ciliogenesis. Specifically, interfering with MAPK15 functions reduces HH signaling and assembly of primary cilia both in immortalized NIH3T3 mouse fibroblasts and in transformed human medulloblastoma cells belonging to the SHH-driven subtype. Also, we show that MAPK15 regulates self-renewal of MB stem cell-like cells, suggesting that inhibition of this kinase may reduce the MB cancer stem cell compartment, possibly diminishing the malignant potential of these tumors. Ultimately, we provide direct evidences that MAPK15 effects on the HH pathway specifically depend on the ability of the kinase of disturbing ciliary structures as effects of activating mutation of the ciliary-independent GLI2 transcription factor are not affected by downregulation of MAPK15 expression. Based on the scarce therapeutic options available for medulloblastoma patients, our data support the possibility of exploiting novel anti-tumoral pharmacological approaches targeting this often-underestimated MAP kinase.

## 2. Materials and Methods

### 2.1. Cell Lines

NIH3T3 (CRL-1658), IMCD3 (CRL-2123), RPE1 (CRL-4000), and DAOY (HTB-186) cells were purchased from ATCC. UW228 and ONS-76 cell lines were kindly provided by Dr. Charles G. Eberhart (John Hopkins University, Baltimora, MD, USA) with the agreement of Dr. Mike Bobola (University of Washington, Seattle, WA, USA). NIH3T3, IMCD3, and RPE1 cells were cultured in Dulbecco’s Modified Eagle Medium (DMEM) (Euroclone, Milano, Italy) supplemented with 10% heat-inactivated fetal bovine serum (FBS), 1% glutamine, and 1% penicillin/streptomycin at 37 °C with 5% CO_2_. DAOY (3 × 10^4^/mL) were cultured in MEM/EBSS supplemented with 10% heat-activated fetal bovine serum, sodium pyruvate, non-essential amino acids (NEAA), 2 mL-Glutamine, and 100 g/mL streptomycin and 100 U/mL penicillin; UW228 (5 × 10^4^/mL) were cultured in DMEM/F12 supplemented with 10% heat-activated fetal bovine serum, 2 mL-Glutamine, 100 g/mL streptomycin, and 100 U/mL penicillin; and finally, ONS-76 were cultured (3 × 10^4^/mL) in RPMI supplemented with 10% heat-activated fetal bovine serum, NEAA, 2 mL-Glutamine, 100 g/mL streptomycin, and 100 U/mL penicillin. Serum concentrations were reduced to 1% during experiments for all cell types, if not otherwise stated. All cell lines were regularly tested by PCR for potential Mycoplasma contamination. Transduced cells were selected with puromycin (Invivogen, San Diego, CA, USA). Ro-318220 (VWR International, Radnor, PA, USA) was dissolved in DMSO.

### 2.2. Plasmids and Lentiviral Vectors

Lentiviruses for gene silencing and overexpression were produced in HEK-293T by co-transfecting lentiviral vector, dR8.74 packaging plasmid (Addgene #22036) and pMD2.G envelope plasmid (Addgene #12259). Plasmids used for overexpression were: pcw107 (empty vector) (Addgene #62511), human SMO-M2 W535L-pcw107-V5 (Addgene, #64628), and human GLI2-ΔN -pcw107-V5 (Addgene, #64626). Vectors used for silencing were: shSCR (scrambled control), shMAPK15_#00 (GCAATGGAATCTCTGCACG), and shMAPK15_#01 (CTGCTCTGCACTAAGGCGC) targeting mouse MAPK15; and shMAPK15_#46 (CACTGACTTCCTCCAATAA) and shMAPK15_#48 (CAGAGAACATTCCGGGAAA) targeting human MAPK15.

### 2.3. Luciferase Reporter Assay

A GLI-responsive luciferase reporter (8 × 3′GLI-BS) (kind gift from H. Sasaki) [23] was used in combination with Renilla luciferase pRL-TK reporter vector (Promega, Madison, WI, USA) (ratio 10:1) to normalize luciferase activity as already described [24]. Luminescence was quantified using the Dual-Glo Luciferase Assay System (Promega) and the GloMax 20/20 Luminometer (Promega, Madison, WI, USA). SAG (#AG-CR1-3585-M005, Adipogen, San Diego, CA, USA) was used at indicated concentrations for 48 h in low-serum conditions.

### 2.4. Medullo-Spheres

DAOY and ONS-76 medullo-spheres were cultured in human embryonic stem cell medium supplemented with 4 ng/mL basic fibroblast growth factor (bFGF), as previously reported [24,25]. For primary sphere formation, cells were plated in 12-well plates (Corning, New York, NY, USA) at 2.5 cell/μL dilution, allowed to form non-adherent spheres and counted after 7 days. For self-renewal assay, primary spheres were dissociated into single cells and re-plated at 2.5 cell/μL dilution in ultra-low adherent 12-well plates. After 1 week, secondary spheres were photographed and counted with a LEICA (Wetzlar, Germany) DFC450C microscope with 4X objective lens, and both length and width of each sphere were measured using Image J.

### 2.5. Western Blotting

Cells were lysed in cold RIPA buffer (50mM Tris-HCl pH 7.5, 1% NP-40, 150mM NaCl, 5mM EDTA, 0.25% NaDOC, and 0.1% SDS) supplemented with protease and phosphatase inhibitors and centrifuged at 20,000× *g* for 20 min at 4°C [26]. Supernatant was collected as whole cell extract (WCE). Equal amounts of protein were resolved by SDS-polyacrylamide gel electrophoresis, transferred onto nitrocellulose membranes, and incubated for 1 h in blocking buffer at room temperature. The following primary antibodies were used: mouse anti-GLI1 (#2643, Cell Signaling Technologies, Danver, MA, USA), mouse anti-SMO (sc-166685, Santa Cruz Biotechnology, Dallas, TX, USA), and mouse anti-HSP90α/β (sc-13119, Santa Cruz Biotechnology, Dallas, TX, USA). After incubation with the corresponding horseradish peroxidase (HRP)-coupled secondary antibody, membranes were developed by using SuperSignal West Femto (Thermo Fisher Scientific, Waltham, MA, USA) and imaged with ChemiDoc Imaging Systems (Bio-Rad, Hercules, CA, USA).

### 2.6. Immunofluorescence

Cells were washed with PBS and next fixed with ice-cold methanol for 10 min. Then, cells were washed three times in PBS and subsequently permeabilized with 0.2% Triton X-100 solution for 10 min. Cells were blocked for 30 min with a 1% BSA (Sigma-Aldrich, St. Louis, MO, USA) in PBS. After this time, cells were incubated with ARL13B (Proteintech, Rosemont, IL, USA) primary antibody for 1 h, washed three times, and then incubated with appropriate Alexa Fluor secondary antibodies; subsequently, cells were washed three times. Nuclei were stained with a solution of 6 μM of 4′,6-diamidino-2-phenylindole (DAPI; Sigma Aldrich, St. Louis, MO, USA) in PBS for 10 min. Coverslips were mounted in fluorescence mounting medium (S3023, Dako, Glostrup, Denmark). Samples were visualized on a TSC SP5 confocal microscope (Leica, Wetzlar, Germany) installed on an inverted LEICA DMI 6000CS (Leica, Wetzlar, Germany) microscope and equipped with an oil PlanApo 40× 1.25 NA objective. Images were acquired using the LAS AF acquisition software (Leica, 10210).

### 2.7. Transient Knock-Down of Endogenous MAPK15

MAPK15-specific siRNA (MAPK15; target sequence 5′-TTGCTTGGAGGCTACTCCCAA-3′) and control non-silencing siRNA (Scramble, target sequence 5′-AATTCTCCGAACGTGTCACGT-3′) were obtained from Qiagen. All siRNAs were transfected at a final concentration of 100 nM using RNAiMAX (Thermo Fisher Scientific, Waltham, MA, USA). Samples were collected 72 h after transfection.

### 2.8. Cell Count

Briefly, cells were seeded in 12-well plates at 3 × 10^4^ cells per well, in triplicate. Then, cells were harvested at indicated times and the cell number were determined using the Z2 Coulter Counter (Beckman Coulter, Brea, CA, USA).

### 2.9. Statistical Analysis

Data represent mean ± SD or mean ± SEM values calculated on at least three independent experiments. *p* values were calculated using Student’s t-test (two groups) or one-way analysis of variance (ANOVA) with Tukey test (more than two groups). A two-tailed value of *p* < 0.05 was considered statistically significant. *, *p* < 0.05; **, *p* < 0.01, ***, *p* < 0.001; ns, not significant.

## 3. Results

### 3.1. MAPK15 Controls Primary Ciliogenesis in A Kinase-Dependent Fashion

**Figure 1:**
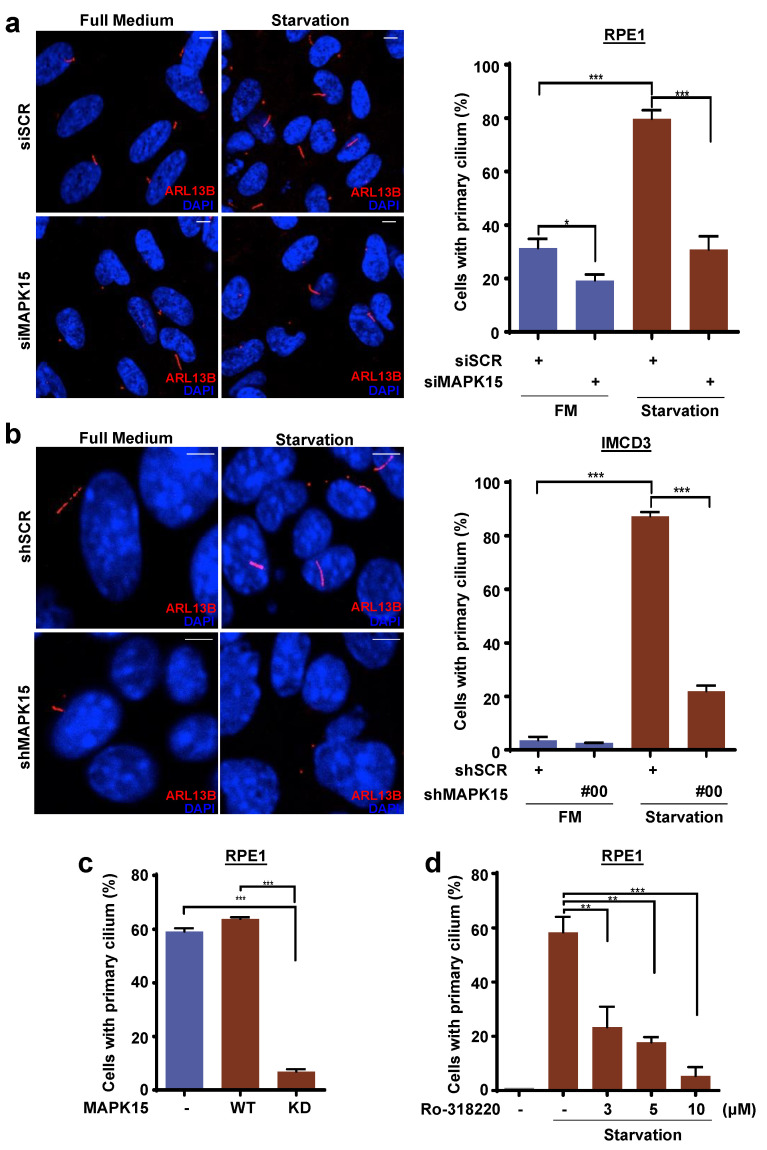
MAPK15 controls primary ciliogenesis in a kinase-dependent fashion. (**a**) RPE1 cells were transfected with scrambled siRNA (siSCR, negative control) or MAPK15 siRNA (siMAPK15) and, after 24 h, incubated in full medium (FM) or starvation media (starvation) for an additional 48 h. Cells were next fixed and subjected to immunofluorescence analysis. In these representative images, ARL13B is visualized in red and nuclei in blue (DAPI). Scale bars correspond to 25 μm. Percentage of primary cilium-positive cells was plotted on the accompanying graph (right panel). (**b**) IMCD3 cells, stably expressing scrambled (shSCR) or MAPK15 (shMAPK15)-specific shRNAs were incubated in full medium (FM) or starvation media for an additional 24 h. Cells were next fixed and subjected to immunofluorescence analysis. In these representative images, ARL13B is visualized in red and nuclei in blue (DAPI). Scale bars correspond to 25 μm. Percentage of primary cilium-positive cell was plotted on the accompanying graph (right panel). (**c**) RPE1 cells were transfected with empty vector (Ctrl), MAPK15_WT, or MAPK15_KD, and, after 24 h, incubated in starvation media for an additional 24 h. Cells were next fixed and subjected to immunofluorescence analysis to score the number of cilium-positive cells. Percentage of primary cilium-positive cell was plotted on the graph. (**d**) RPE1 cells were incubated in starvation media and treated with indicated amounts of the Ro-318220 MAPK15 inhibitor for 24 h. Cells were next fixed and subjected to immunofluorescence analysis to score the number of cilium-positive cells. Percentage of primary cilium-positive cell was plotted on the graph. One experiment, which is representative of three independent experiments, is shown in the different panels (*n* = 3). *, *p* < 0.05; **, *p* < 0.01, ***, *p* < 0.001.

MAPK15 was reported to localize to the basal bodies of motile [8] and primary cilia [7] in vertebrates and to regulate their formation. Indeed, interfering with MAPK15 expression in both RPE1 (Figure 1a and Figure S1a) and IMCD3 (Figure 1b and Figure S1b) cells, two established models for studying primary ciliogenesis [27,28], respectively in human and murine cells, strongly inhibited their ability of assembling primary cilia. Interestingly, Kazatskaya et al. recently showed that a MAPK15 kinase-dead mutant was not able to rescue siRNA-dependent inhibition of primary cilia formation in RPE1 cells [7], suggesting a key role for MAPK15 kinase activity in regulating this process. We therefore decided to confirm and expand this concept by demonstrating that the MAPK15 kinase dead (KD) mutant is able, by itself, to inhibit primary ciliogenesis in these cells (Figure 1c) and that also pharmacological inhibition of MAPK15 activity by one of its described drug inhibitors, Ro-318220 [13], was able to prevent cilia assembly in RPE1 cells (Figure 1d). Overall, these and previous data support a role for MAPK15 kinase activity in primary ciliogenesis in mammalian cells.

### 3.2. MAPK15 Regulates the HH Pathway in NIH3T3 Cells

**Figure 2:**
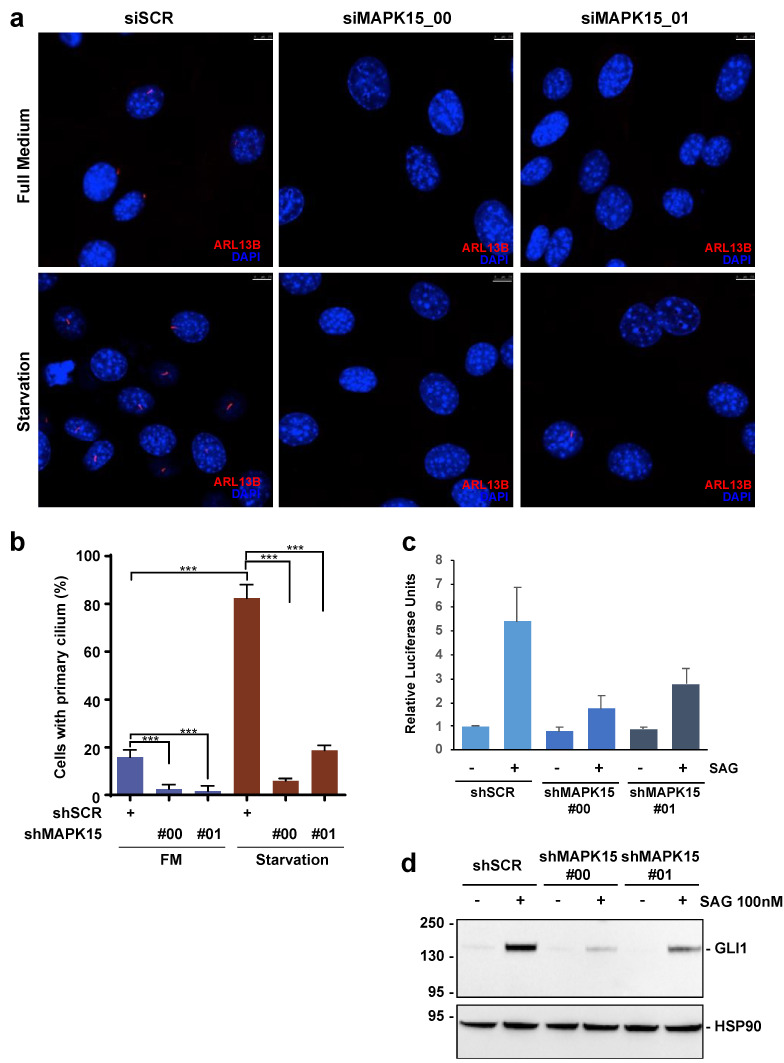
MAPK15 regulates the HH pathway in NIH3T3 cells. (**a**) NIH3T3 cells, stably transduced for scrambled (shSCR) or two different MAPK15 (shMAPK15)-specific shRNAs were incubated in full medium (FM) or starvation media for an additional 24 h. Cells were next fixed and subjected to immunofluorescence analysis. In these representative images, ARL13B is visualized in red and nuclei in blue (DAPI). Scale bars correspond to 7.5 μm. Percentage of primary cilium-positive cell was plotted on the accompanying graph (**b**). (**c**) Quantification of GLI-dependent luciferase reporter assay in HH-responsive NIH3T3 cells stably transduced for shSCR, shMAPK15_#00, or shMAPK15_#01 and treated with 100 nM SAG for 48 h. Relative luciferase units were GLI-dependent reporter firefly/renilla control ratios, with untreated cells equated to 1. (**d**) Western blot of endogenous Gli1 protein in NIH3T3 cells transduced as indicated and treated with 100 nM SAG for 48 h. HSP90 was used as loading control. ***, *p* < 0.001. The uncropped blots are shown in supplementary materials (Figure S6).

The primary cilium is important to compartmentalize several cellular signals controlling animal development, among which are Wingless, PDGFα, SHH, and NOTCH [29]. Importantly, localization into this organelle of proteins belonging to these pathways has been already shown to regulate their signals, ultimately preventing or altering their transmission when cilia are not properly formed [29,30]. Based on the role of MAPK15 in primary cilia biogenesis, we, therefore, decided to investigate a potential role for this kinase in HH signaling. At this scope, we first tested MAPK15-dependent primary cilia formation in NIH3T3 cells, which is a well-established model for the study of both the HH pathway [25] and of ciliogenesis [27]. To modulate MAPK15 expression in these cells, we decided to use the stable expression of shRNAs for this kinase (Figure S2) and demonstrated that this approach strongly reduced starvation-dependent primary ciliogenesis in these cells (Figure 2a,b). In these cellular settings, we next stimulated the HH pathway using the Smoothened AGonist (SAG), which acts as an activator of the G protein-coupled receptor (GPCR) SMO [31], and tested HH pathway activation upon downregulation of MAPK15 expression. Interestingly, knock-down of this kinase by two independent shRNAs significantly reduced transcriptional activation of a GLI-responsive luciferase reporter (Figure 2c) and, similarly, protein expression of endogenous GLI1 (Figure 2d), the best read-out of an active HH pathway [25]. Overall, these results therefore demonstrate a role for MAPK15 in modulating the canonical HH signaling, which is possibly related to its role in primary ciliogenesis, with a mechanism similar to that already demonstrated for other cilia-related genes, i.e., KIF3A [21,22] and ARL13B [32].

### 3.3. MAPK15 Controls Hedgehog Signaling in Human Medulloblastoma Cells

**Figure 3:**
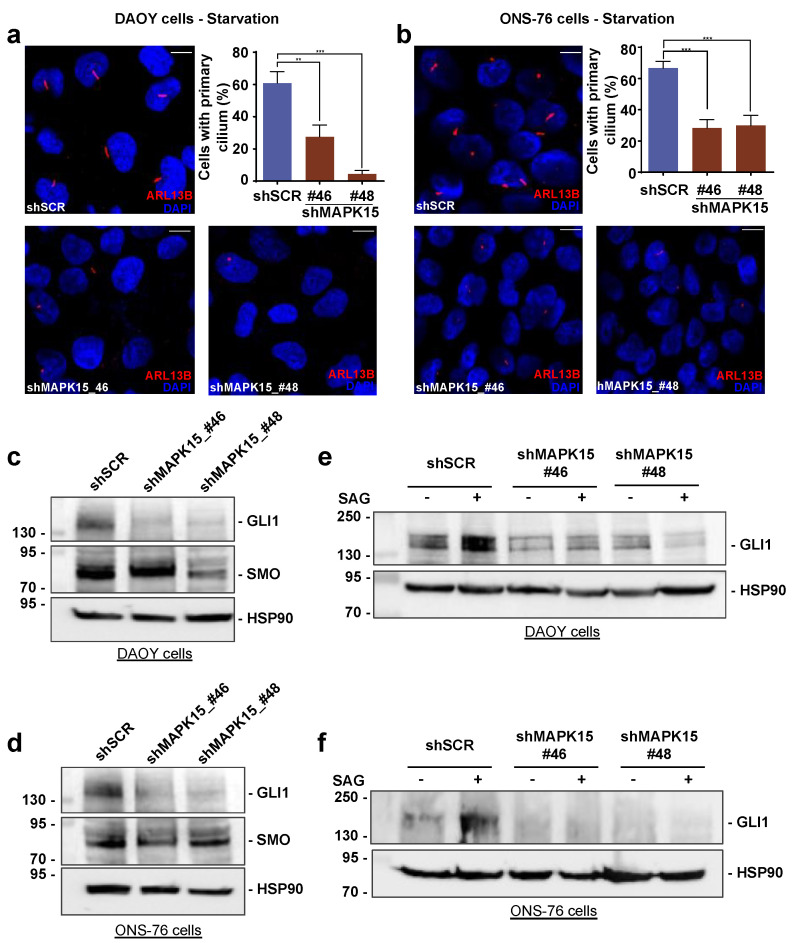
MAPK15 regulates the HH pathway in medulloblastoma cells. DAOY cells (**a**) and ONS-76 cells (**b**) were stably transduced with scrambled (shSCR, negative control) or two different MAPK15 shRNA (shMAPK15_46 or shMAPK15_48) and then analyzed by immunofluorescence, for induction of primary ciliogenesis, upon starvation for 24 h. In these representative images, ARL13B is visualized in red and nuclei in blue (DAPI). Scale bars correspond to 25 μm. Percentage of primary cilium-positive cell was plotted on the accompanying graphs (upper right panel). (**c**–**f**) Representative Western blot of GLI1 in DAOY (**c**) and ONS-76 (**d**) stably transduced with scramble (shSCR), shMAPK15_46, or shMAPK15_48. HSP90 was used as loading control. (**e**,**f**) Western blot of GLI1 in DAOY (**e**) and ONS-76 (**f**) showing that silencing of MAPK15 prevents the increase of GLI1 protein levels upon SAG stimulation. **, *p* < 0.01, ***, *p* < 0.001. The uncropped blots are shown in supplementary materials (Figure S6).

Mutations affecting proteins involved in HH signaling, including loss of the negative regulator PTCH1 and the activating point mutation in SMO (W535L for human SMO and W539L for mouse Smo), result in hyperactivation of the HH pathway and can lead to cilia-dependent tumor formation in MB [33]. Indeed, MB cells represent a key model for investigating the effect of canonical HH signaling in tumor development, particularly regarding its role in cancer stem cells (regarded as brain tumor-initiating cells). Among such cellular models, DAOY and ONS-76 are well-known MB cell lines and are considered representative of primary tumors [34]. Importantly, both these cells belong to the SHH subtype of medulloblastomas, but differ because DAOY have a mutation in p53 while ONS-76 are wild-type for this gene [35]. Thus, we decided to investigate the role of MAPK15 in these cells, by a stable knockdown approach. Indeed, when interfering in MAPK15 expression (Figure S3), both DAOY (Figure 3a) and ONS-76 (Figure 3b), cells strongly reduced their ability to assemble primary cilia. Consequently, both cell types showed reduced levels of endogenous GLI1 upon MAPK15 down-regulation (Figure 3c,d). Ultimately, SAG stimulation failed to increase GLI1 protein levels in both cell types, when interfering in MAPK15 expression (Figure 3e,f), overall demonstrating a specific requirement for MAPK15 activity to properly assemble primary cilia and signal through the canonical HH pathway.

### 3.4. MAPK15 Regulates Self-Renewal In Vitro of Medulloblastoma Stem Cell-Like Cells

**Figure 4:**
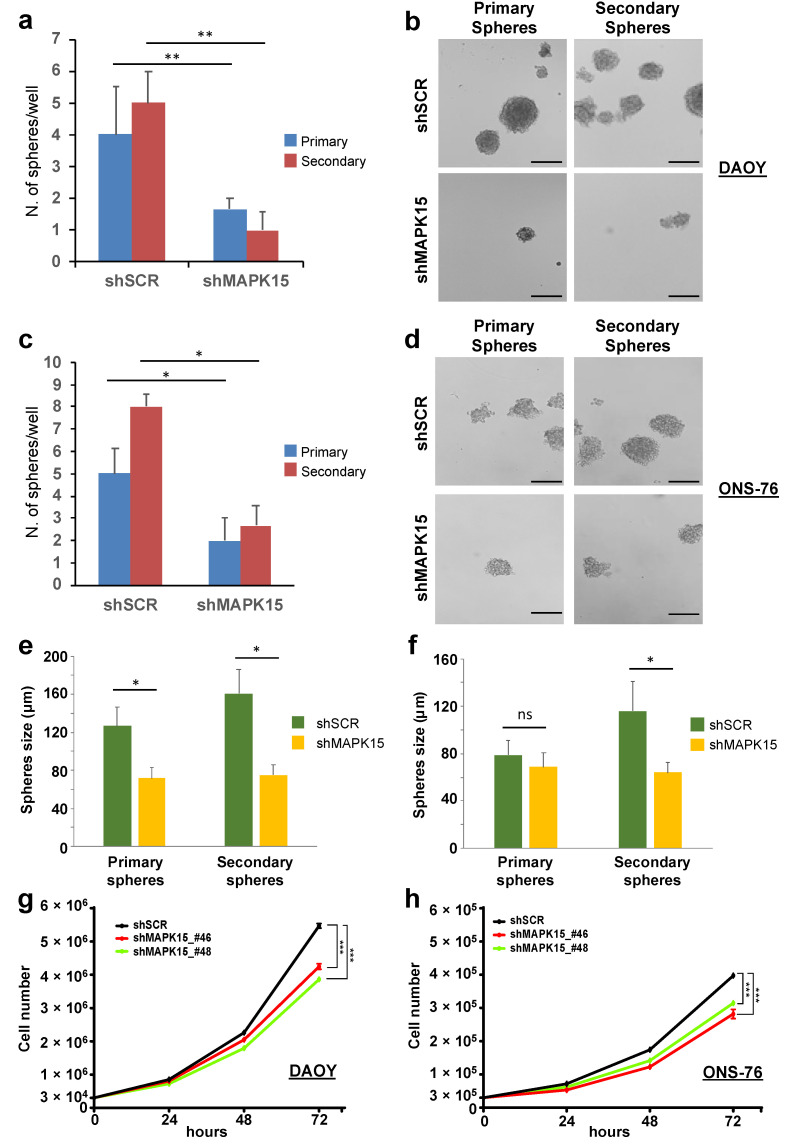
MAPK15 controls the enrichment of the medulloblastoma stem cell compartment. Number (**a**,**c**) and representative images (**b**,**d**) of primary and secondary spheres in DAOY (**a**,**b**) and ONS-76 (**c**,**d**) cells stably transduced with scrambled (shSCR) or MAPK15 (shMAPK15) shRNAs. (**e**,**f**) Size of primary and secondary DAOY (**e**) and ONS-76 (**f**) spheres transduced, as indicated. Scale bar = 150 μm. DAOY (**g**) and ONS-76 (**h**) cells (3 × 10^4^), stably transduced with scrambled (shSCR, negative control) or two different MAPK15 shRNA (shMAPK15_#46 or shMAPK15_#48), were seeded in 12-well plates and then harvested at indicated time-points (24, 48 and 72 h) and counted. *, *p* < 0.05; **, *p* < 0.01; ***, *p* < 0.001; ns, not significant.

A subpopulation of cancer cells with stem-like features, referred to as cancer stem cells (CSC) or tumor-initiating cells, has been identified and characterized by several groups in MBs [36,37]. We already described medullo-spheres from DAOY and ONS-76 cells as a model to study CSC in vitro for evaluating progression and malignancy of medulloblastomas and demonstrated higher expression of β-catenin and Sox-2 in medullo-spheres compared to MB adherent cells [34]. As the tumor-sphere assay allows the enrichment of potential MB-initiating cells, we compared MAPK15 expression in adherent cells and in spheres derived from DAOY, UW228, and ONS-76 cells, by qPCR. Interestingly, we found that medullo-spheres obtained from all these cells were greatly enriched in the expression of MAPK15 mRNA, when compared with the corresponding adherent cells (Figure S4). Based on the increased expression of MAPK15 in the cancer stem cell-enriched population of MB, we next evaluated whether the kinase was involved in regulating self-renewal of putative MB-initiating cells. To test this possibility, we measured the ability of DAOY and ONS-76 cells to form primary and secondary spheres after MAPK15 silencing. Interestingly, ablation of MAPK15 significantly reduced primary sphere formation and their ability to self-renew and form secondary spheres from single cell suspension of primary spheres in both DAOY (Figure 4a,b) and ONS-76 (Figure 4c,d) cells. Interestingly, we noticed that primary and secondary spheres depleted for MAPK15 were also reduced in size, especially those obtained from DAOY cells (Figure 4e,f). This suggests that silencing of MAPK15, besides reducing CSC self-renewal in vitro, negatively affects also proliferation and/or survival of more differentiated neural progenitors that constitute the bulk of tumor-spheres. Importantly, the observed effect of MAPK15 down-regulation on the establishment of tumor spheroids was not due to an impediment of tridimensional cell aggregation, as a significant reduction in MB cell proliferation was also observed in bidimensional monolayer cultures upon knock-down of the endogenous gene (Figure 4g,h), supporting the idea that targeted inhibition of MAPK15 activity is potentially exploitable in MB patients and may positively affect the prognosis of this disease.

### 3.5. MAPK15 Effects on the Hedgehog Pathway Depends on the Ability of the Kinase of Affecting Ciliary Structures

**Figure 5:**
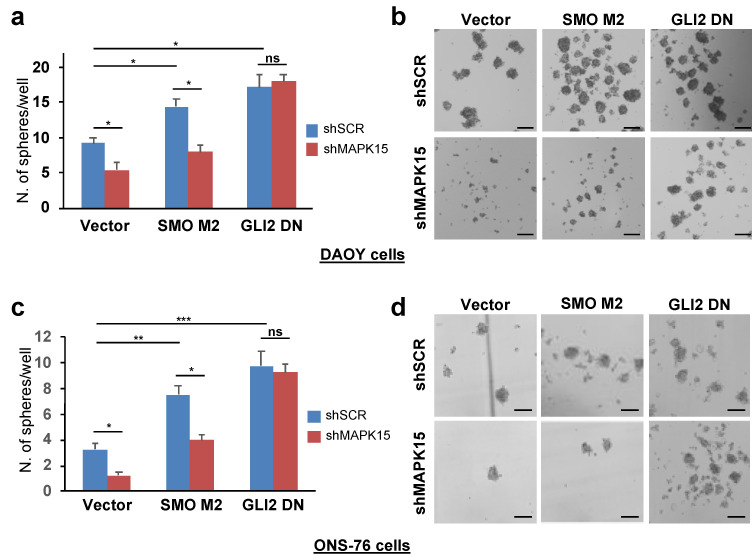
Overexpression of GLI2-ΔN, but not of SMO-M2, rescues the decrease in medullo-sphere self-renewal upon MAPK15 silencing. Number (**a**,**c**) and representative images (**b**,**d**) of secondary spheres in DAOY (**a**,**b**) and ONS-76 (**c**,**d**) cells transduced with shSCR (blue bars) or shMAPK15 (red bars), and transduced with empty vector, SMO-M2, or GLI2-ΔN. Scale bar = 150 μm. *, *p* < 0.05; **, *p* < 0.01, ***, *p* < 0.001; ns, not significant.

Previous data suggested that primary cilia can be either permissive or inhibitory for MB or BCC formation, depending on the underlying oncogenic events [21,22]. Specifically, in MB, genetic ablation of primary cilia blocked tumor formation when this was driven by a constitutively active SMO, stimulating canonical HH activation, but increased transformation when driven by a constitutively active GLI2 protein, a transcription factor acting “outside” and downstream the primary cilium [21]. We, therefore, reasoned that if the observed MAPK15 effects on HH signaling were due to inhibition of ciliogenesis, they should not affect the activity of the oncogenic GLI2-ΔN, a truncated GLI2 protein lacking the 328 N-terminal amino acids, which is endowed with enhanced transcriptional activity compared to full length GLI2 [38]. Indeed, in both DAOY (Figure 5a,b) and ONS-76 (Figure 5c,d) cells, the increase in medullo-sphere self-renewal induced by GLI2-ΔN overexpression was not affected by MAPK15 silencing while ablation of MAPK15 reduced self-renewal ability induced by SMO-M2 overexpression. Altogether, these results suggest that MAPK15 acts upstream of GLI2 in regulating CSC self-renewal, by controlling the formation of the ciliary compartment where canonical HH signaling is initiated.

### 3.6. Inhibition of MAPK15 Kinase Activity by Ro-318220 Prevents Cell Proliferation of SHH-Driven Medulloblastoma Cells

**Figure 6:**
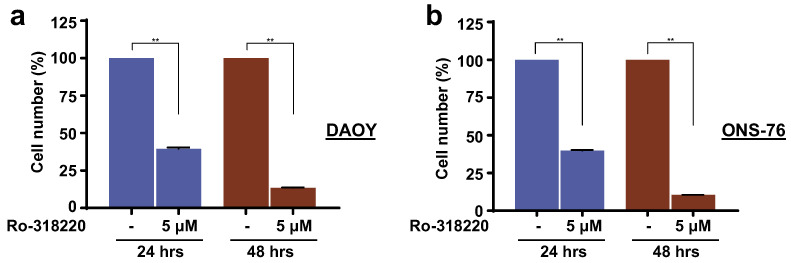
MAPK15 pharmacological inhibition reduces medulloblastoma cell proliferation. DAOY (**a**) and ONS-76 (**b**) cells (3 × 10^4^) were seeded in 12-well plates and, after 24 h, treated with the Ro-318220 MAPK15 inhibitor (5 μM), for the indicated times. Cells were next harvested and counted. **, *p* < 0.01.

Our findings overall suggest that inhibition of MAPK15 activity by specific pharmacological compounds could, potentially, be used to treat MB by blocking cilia formation. To test this hypothesis, we therefore decided to evaluate proliferation of DAOY and ONS-76 cells upon treatment with a specific MAPK15 inhibitor. We indeed treated MB cells with the Ro-318220 compound and showed that it exerted an efficient antiproliferative effect in these cells (Figure 6a,b), overall suggesting this kinase as a potential new actionable molecular target for medulloblastomas but also other tumors with deregulated canonical HH signaling, e.g., basal cell carcinomas [39].

## 4. Discussion

Our data show that MAPK15 knock-down reduces canonical HH signaling and inhibits proliferation of SHH-driven MB cell lines and generation of cancer stem cells. Specifically, MAPK15 down-regulation diminished the HH transcriptional response when the pathway was stimulated, in NIH3T3 cells, with the agonist SAG, acting on the endogenous G protein-coupled receptor (GPCR) SMO, but also in DAOY and ONS-76 MB cells, both representing typical models for investigating the effect of canonical HH signaling in tumor development. Importantly, we observed consistent results in all these cells, indicating that the effect is robust in distinct cell types and is applicable in both mouse and human cells. Similarly, MAPK15 downregulation also inhibited primary ciliogenesis in all tested cellular systems.

Manipulation of different genes (IFT88, KIF3A, or ARL13B) has been already used to demonstrate the possibility of interfering with primary ciliogenesis and, consequently, with cilia-dependent signaling pathways such as HH and PDGFRα, both in vitro and in vivo [21,22,30,32]. Still, the possibility of envisaging a rapid application in human pathologies of these data is low because of the limited “druggability” of cognate proteins. Conversely, although our analysis currently excludes a correlation between MAPK15 expression and clinical outcome of SHH-driven MBs (Figure S5), the role of this kinase in cilia formation and, consequently, in the activation of cilia-dependent pathways such as HH but also PDGFRα or WNT, may be more rapidly challenged, at least in preclinical studies, by using already available pharmacological inhibitors for this kinase. In this regard, we show that an already reported MAPK15 inhibitor, Ro-318220 [13], is able to prevent cilia assembly in RPE1 cells and inhibit proliferation of MB cell lines, supporting future studies aimed at establishing potential for pharmacological approaches to human tumors relying on cilia for their pathogenesis. This may be immediately evident for tumors such as medulloblastomas and basal cell carcinomas that frequently show mutations in either SMO or PTCH1 genes. Still, based on a suggested paracrine role for HH signaling in tumorigenesis [40], disruption of cilia in cells or related parenchyma of different tumors may indirectly affect their growth, increasing the number of tumors potentially amenable to this approach. This is, therefore, predicted to have implications for future therapeutic methodologies based on inhibition of MAPK15, considering the presence of cilia as a useful biomarker for identifying MBs responsive to MAPK15 antagonists. Nonetheless, cilia have also been demonstrated to exert tumor-suppressive activity for oncogenic HH signaling initiated downstream of SMO [21,22]. Indeed, we also show that interfering for MAPK15 expression strongly reduces the ability of an activated form of SMO to support cancer stem cells self-renewal, while failing to affect the same activity induced by the GLI2 oncogene. Given the dual functions of cilia as structures able to mediate both tumor suppression and transformation [21,22], from a therapeutic standpoint, great care will be needed to identify the initiating oncogenic event in MB patients.

Overall, available data therefore suggest that, for clinical applications, it will be important to learn more about the functions of HH and cilia in different tumors, to be able to separate specific subsets of tumors in which this approach may be beneficial from those that paradoxically exacerbate cancer growth. Equally important, the identification of more specific and powerful MAPK15 inhibitors for in vivo use will warrant further investigation.

## 5. Conclusions

Here, we show that MAPK15 activity supports the assembly of primary cilia in model as well as medulloblastoma cell types, allows canonical HH signaling, and allows the enrichment of the cancer stem cell population, which are usually regarded as the medulloblastoma-initiating cells helping progression and malignancy in these tumors. Our data therefore suggest that inhibition of MAPK15 kinase activity may represent a potentially exploitable approach for tumors supported by oncogenic signals originating from the primary cilium compartment.

## Data Availability

The data presented in this study are available on request from the corresponding author.

## Supplementary Materials

The following are available online at https://www.mdpi.com/article/10.3390/cancers13194903/s1, Figure S1: Quantitative PCR (qPCR) analysis to evaluate expression of the MAPK15 gene in human RPE1 or murine IMCD3 cells; Figure S2: Quantitative PCR (qPCR) analysis to evaluate expression of the MAPK15 gene in murine NIH3T3 cells, stably expressing scrambled (shSCR) or two different MAPK15 (shMAPK15)-specific shRNAs. Figure S3: Quantitative PCR (qPCR) analysis to evaluate expression of the MAPK15 gene in human medulloblastoma cells, stably expressing scrambled (shSCR) or two different MAPK15 (shMAPK15)-specific shRNAs. Figure S4: Quantitative PCR to evaluate mRNA expression of the MAPK15 gene in adherent (Adh) or medullo-spheres (Sph) derived from UW228, ONS-76, and DAOY medulloblastoma cells. Figure S5: MAPK15 expression levels do not affect the survival of SHH-driven medulloblastomas. Supplementary Materials and Methods. Figure S6: Original Western Blot images.

## Abbreviations

HH: Hedgehog; MB, Medulloblastoma; MAP, mitogen activated protein; SHH, Sonic hedgehog; PDGF, platelet-derived growth factor; SAG, Smoothened agonist; PTCH1, Patched1; BCC, basal cell carcinomas; GPCR, G protein-coupled receptor; SMO, Smoothened; CSC, cancer stem cells.
