# Supplementary material for: MAPK15 Controls Hedgehog Signaling in Medulloblastoma Cells by Regulating Primary Ciliogenesis
Source: Cancers (Basel). 2021 Sep 29;13(19):4903. doi: 10.3390/cancers13194903 (PMC8508543; doi:10.3390/cancers13194903)
Supplement: Supplementary file 1 [file cancers-13-04903-s001.zip › cancers-1375728-supplementary.pptx]

## Slide 1
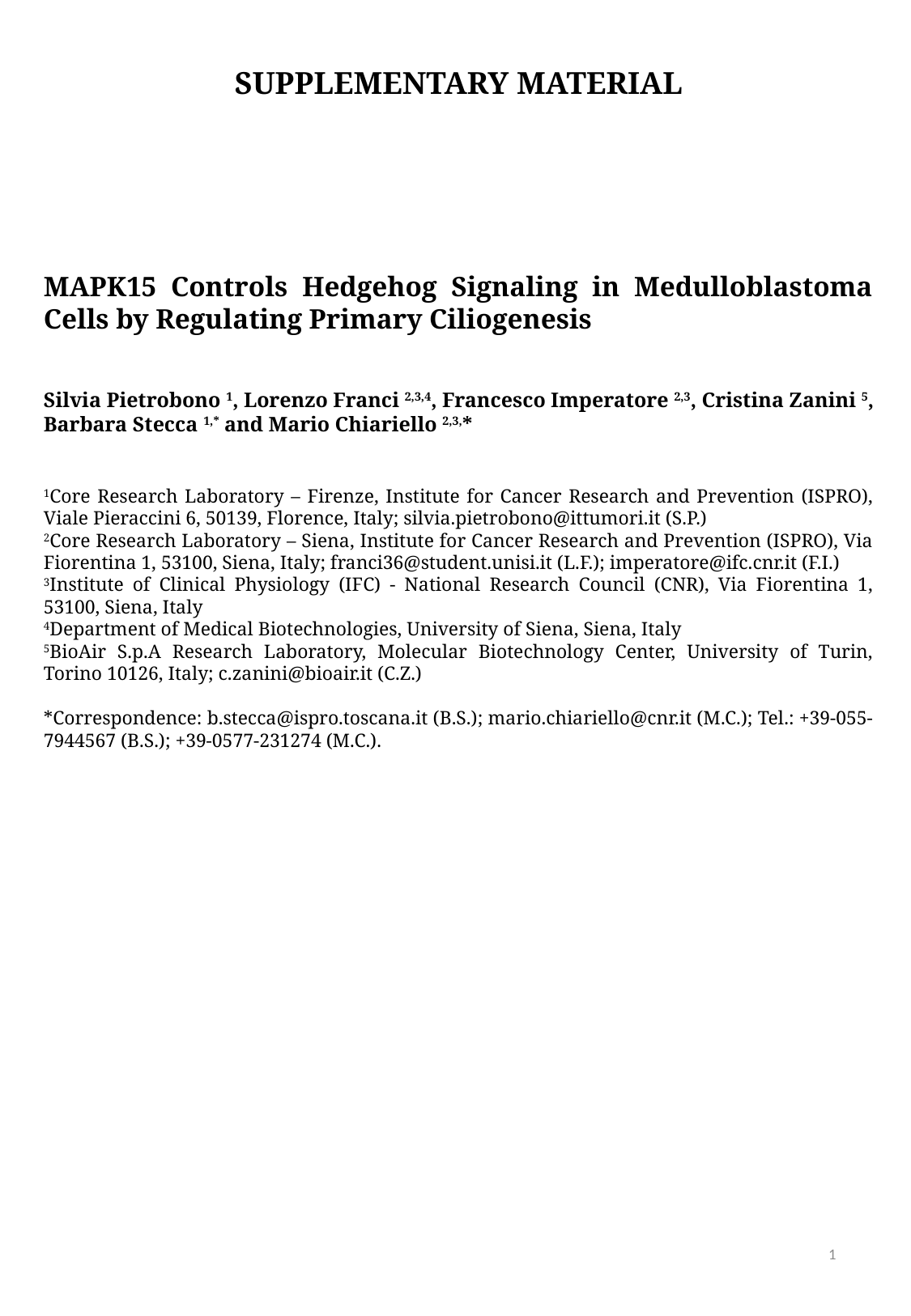

SUPPLEMENTARY MATERIAL
MAPK15 Controls Hedgehog Signaling in Medulloblastoma Cells by Regulating Primary Ciliogenesis
Silvia Pietrobono 1, Lorenzo Franci 2,3,4, Francesco Imperatore 2,3, Cristina Zanini 5, Barbara Stecca 1,* and Mario Chiariello 2,3,*
1Core Research Laboratory – Firenze, Institute for Cancer Research and Prevention (ISPRO), Viale Pieraccini 6, 50139, Florence, Italy; silvia.pietrobono@ittumori.it (S.P.)
2Core Research Laboratory – Siena, Institute for Cancer Research and Prevention (ISPRO), Via Fiorentina 1, 53100, Siena, Italy; franci36@student.unisi.it (L.F.); imperatore@ifc.cnr.it (F.I.)
3Institute of Clinical Physiology (IFC) - National Research Council (CNR), Via Fiorentina 1, 53100, Siena, Italy
4Department of Medical Biotechnologies, University of Siena, Siena, Italy
5BioAir S.p.A Research Laboratory, Molecular Biotechnology Center, University of Turin, Torino 10126, Italy; c.zanini@bioair.it (C.Z.)
*Correspondence: b.stecca@ispro.toscana.it (B.S.); mario.chiariello@cnr.it (M.C.); Tel.: +39-055-7944567 (B.S.); +39-0577-231274 (M.C.).
1

## Slide 2
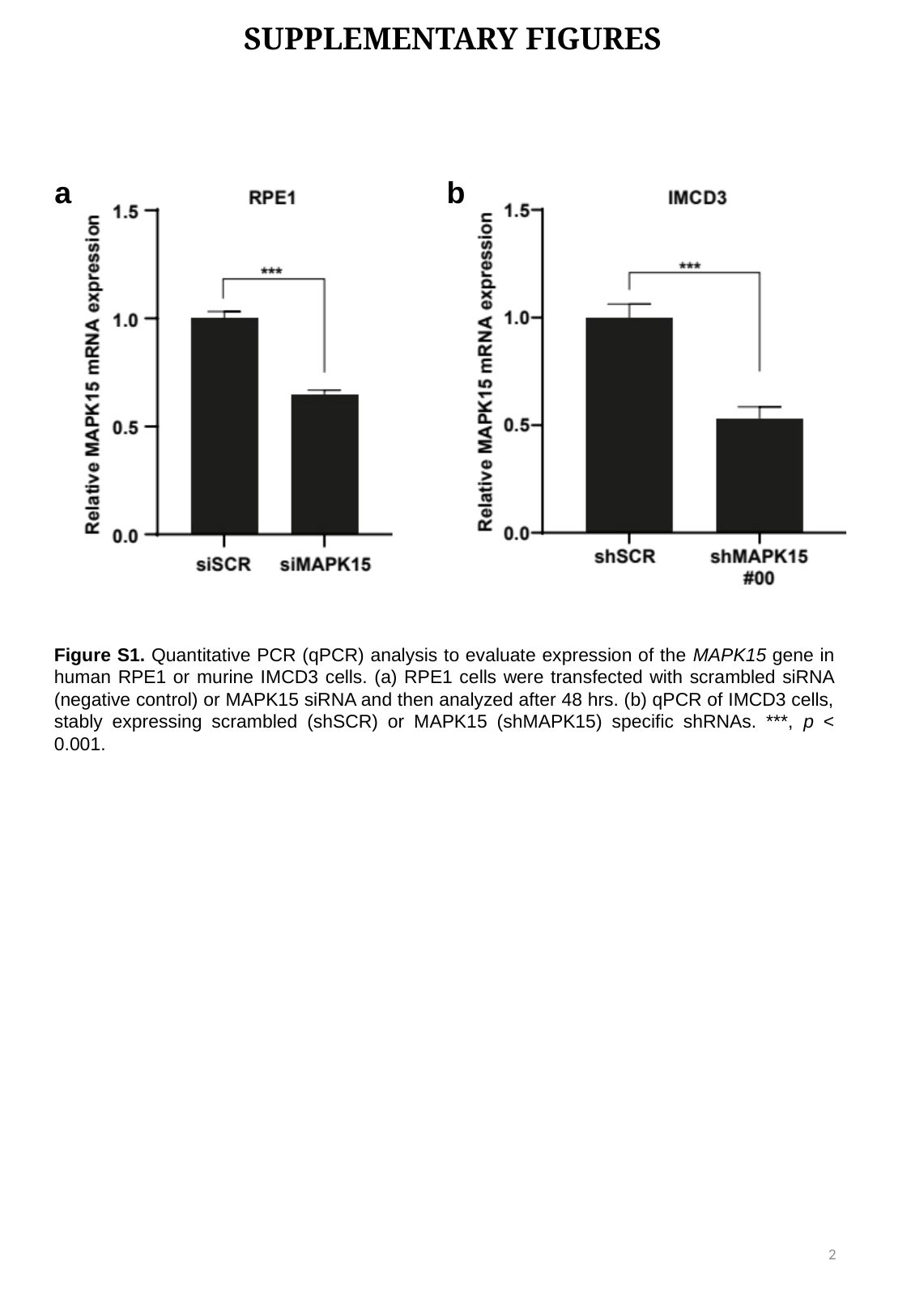

SUPPLEMENTARY FIGURES
a
b
Figure S1. Quantitative PCR (qPCR) analysis to evaluate expression of the MAPK15 gene in human RPE1 or murine IMCD3 cells. (a) RPE1 cells were transfected with scrambled siRNA (negative control) or MAPK15 siRNA and then analyzed after 48 hrs. (b) qPCR of IMCD3 cells, stably expressing scrambled (shSCR) or MAPK15 (shMAPK15) specific shRNAs. ***, p < 0.001.
2

## Slide 3
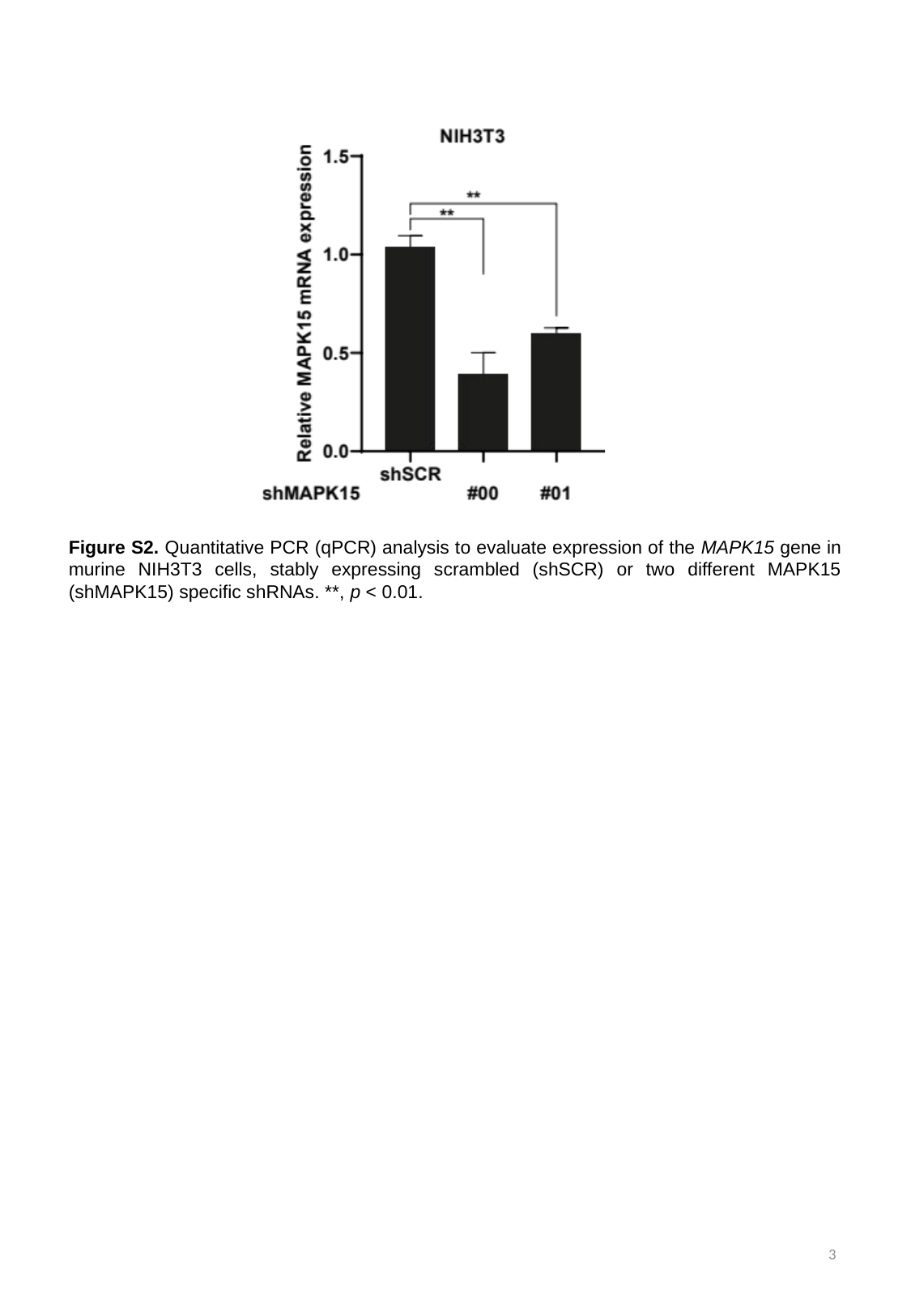

Figure S2. Quantitative PCR (qPCR) analysis to evaluate expression of the MAPK15 gene in murine NIH3T3 cells, stably expressing scrambled (shSCR) or two different MAPK15 (shMAPK15) specific shRNAs. **, p < 0.01.
3

## Slide 4
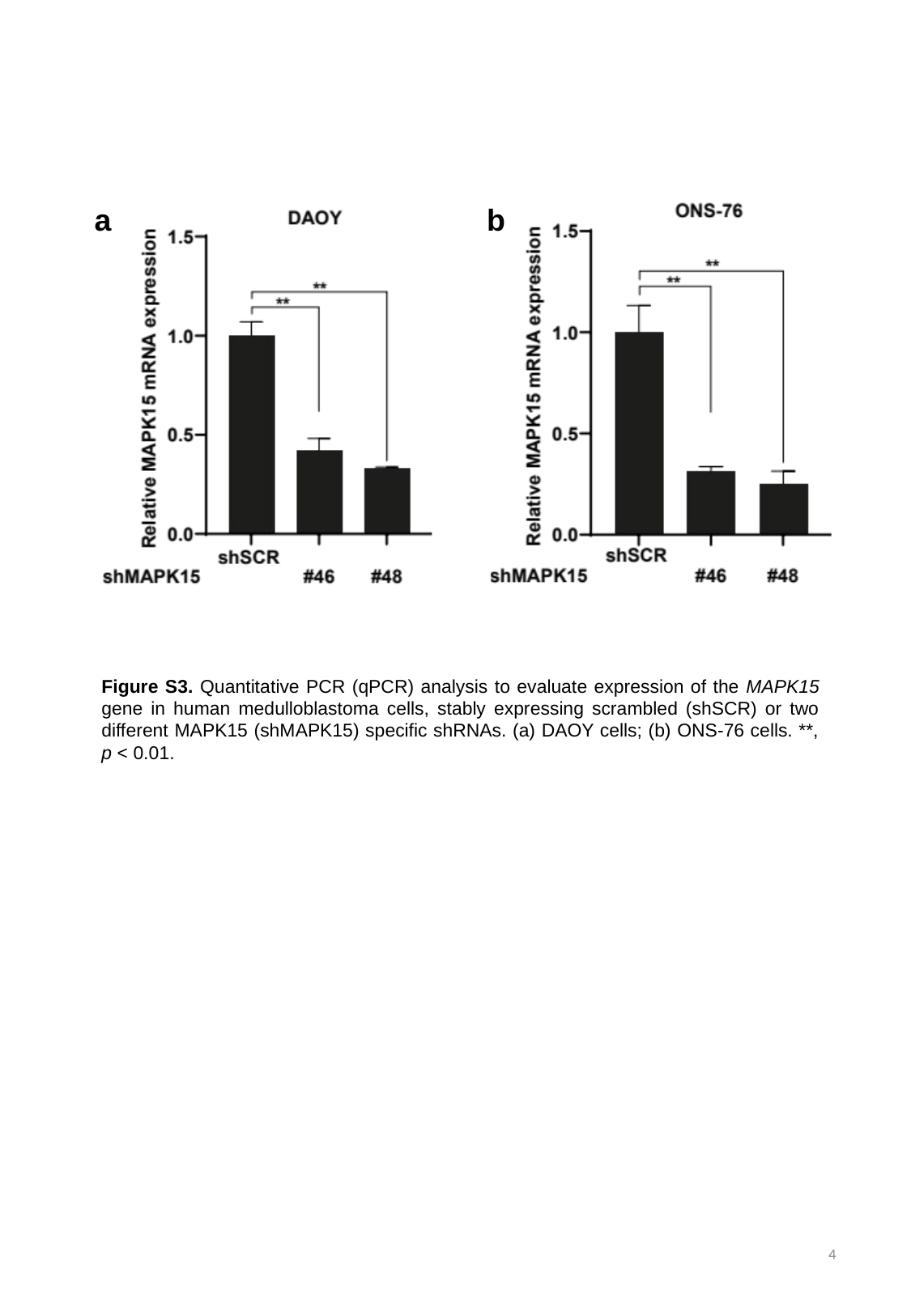

a
b
Figure S3. Quantitative PCR (qPCR) analysis to evaluate expression of the MAPK15 gene in human medulloblastoma cells, stably expressing scrambled (shSCR) or two different MAPK15 (shMAPK15) specific shRNAs. (a) DAOY cells; (b) ONS-76 cells. **, p < 0.01.
4

## Slide 5
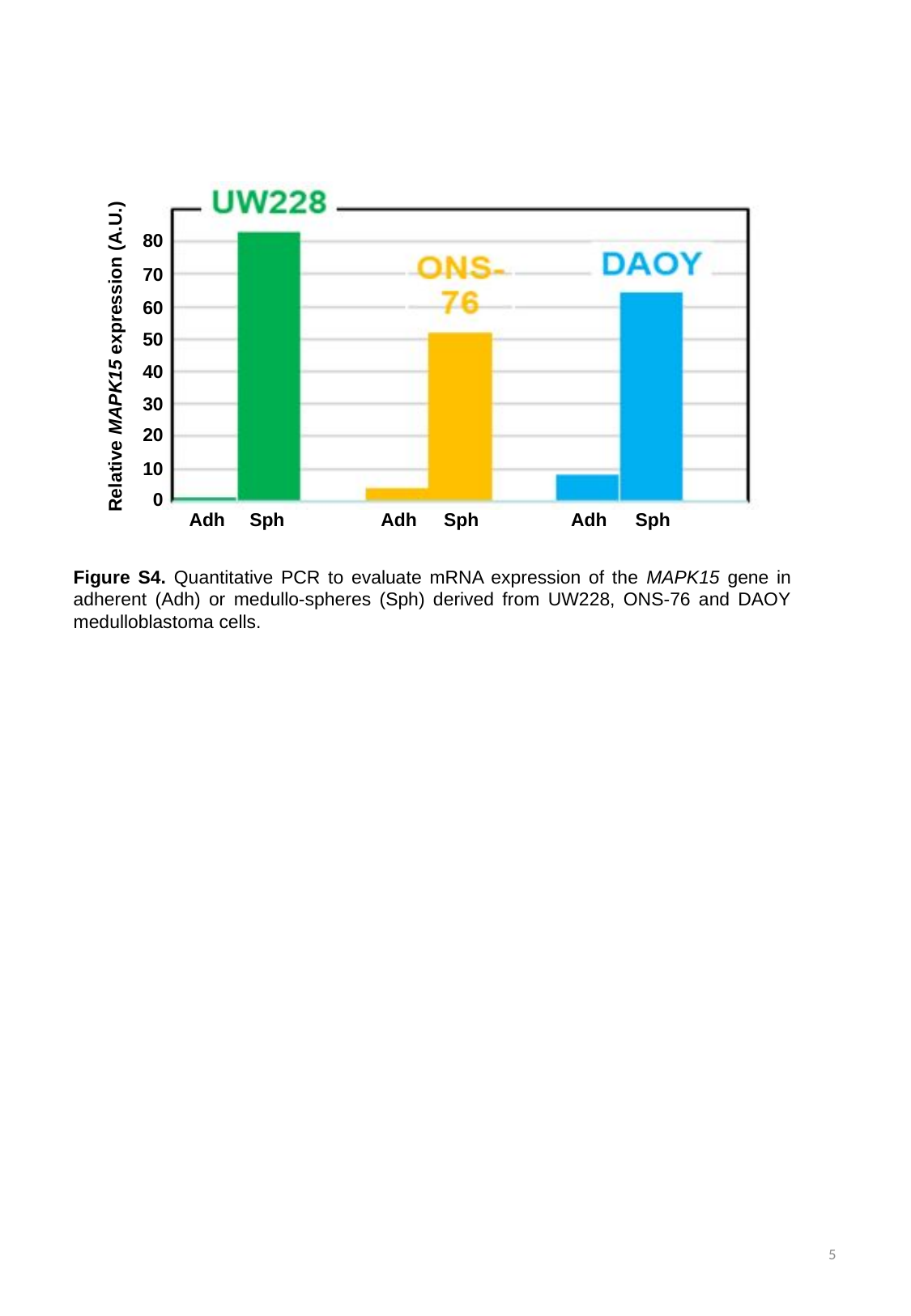

80
70
60
50
Relative MAPK15 expression (A.U.)
40
30
20
10
0
Adh
Sph
Adh
Sph
Adh
Sph
Figure S4. Quantitative PCR to evaluate mRNA expression of the MAPK15 gene in adherent (Adh) or medullo-spheres (Sph) derived from UW228, ONS-76 and DAOY medulloblastoma cells.
5

## Slide 6
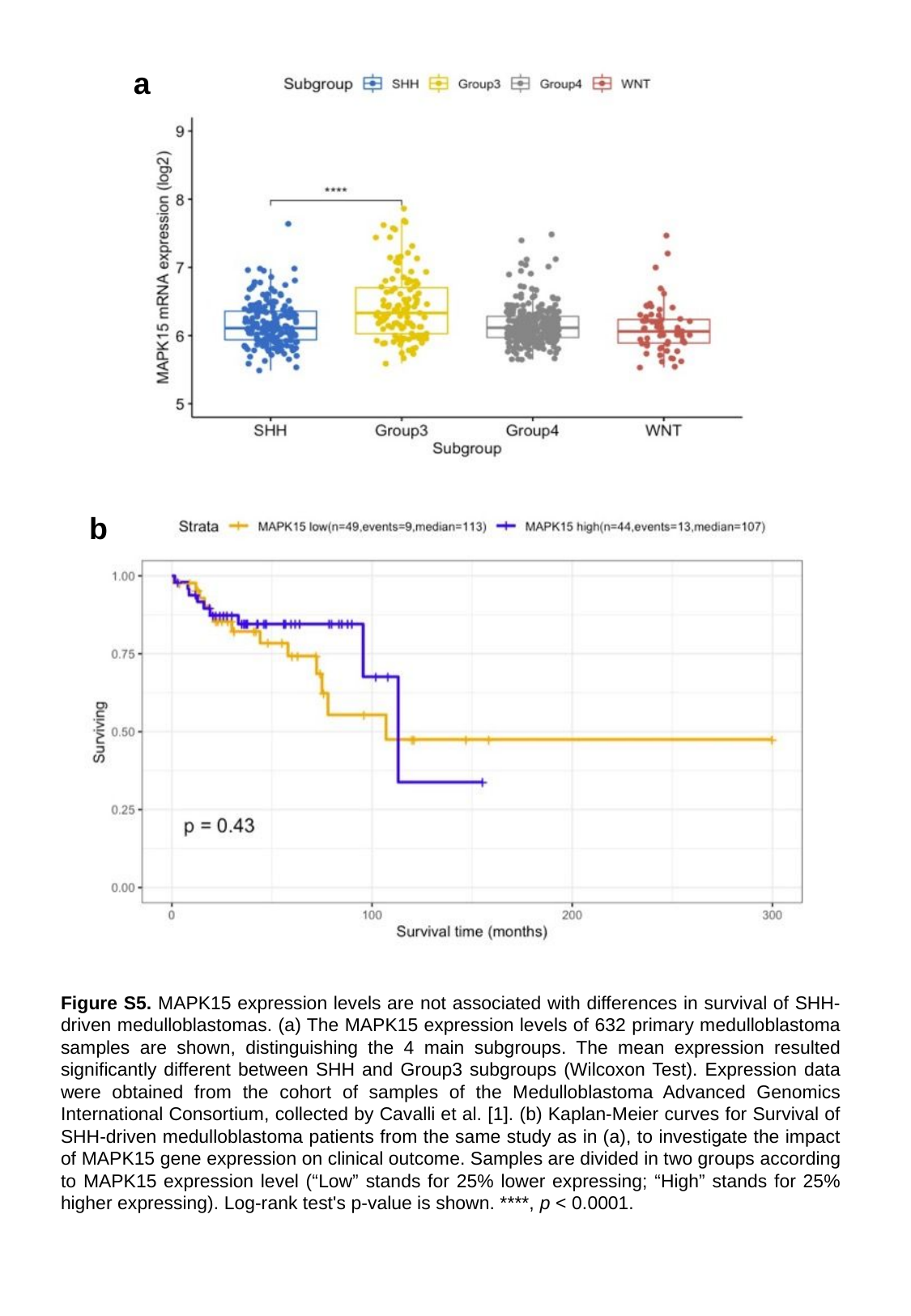

a
b
Figure S5. MAPK15 expression levels are not associated with differences in survival of SHH-driven medulloblastomas. (a) The MAPK15 expression levels of 632 primary medulloblastoma samples are shown, distinguishing the 4 main subgroups. The mean expression resulted significantly different between SHH and Group3 subgroups (Wilcoxon Test). Expression data were obtained from the cohort of samples of the Medulloblastoma Advanced Genomics International Consortium, collected by Cavalli et al. [1]. (b) Kaplan-Meier curves for Survival of SHH-driven medulloblastoma patients from the same study as in (a), to investigate the impact of MAPK15 gene expression on clinical outcome. Samples are divided in two groups according to MAPK15 expression level (“Low” stands for 25% lower expressing; “High” stands for 25% higher expressing). Log-rank test's p-value is shown. ****, p < 0.0001.

## Slide 7
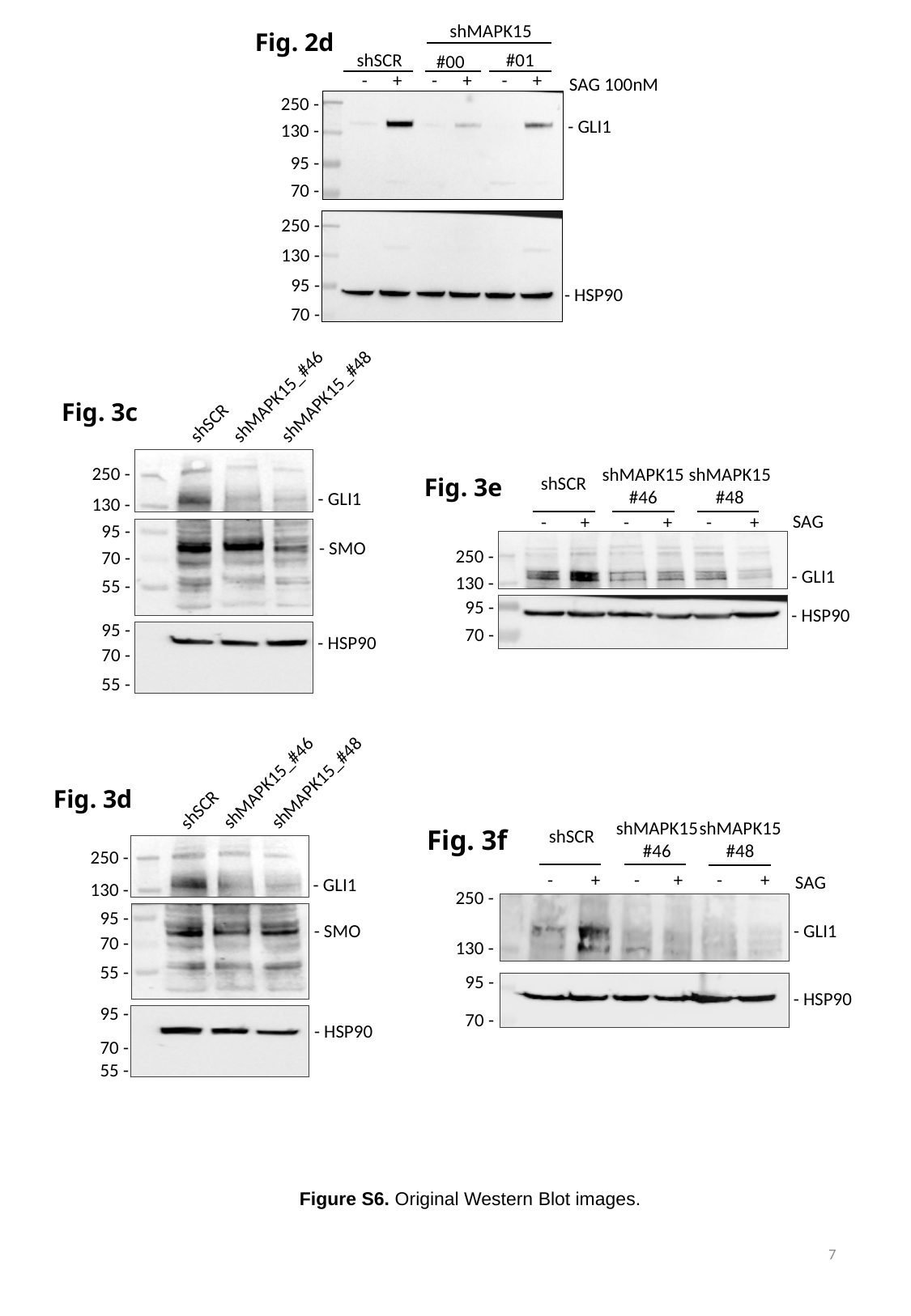

shMAPK15
Fig. 2d
shSCR
#01
#00
- + - + - +
SAG 100nM
250 -
- GLI1
130 -
95 -
70 -
250 -
130 -
95 -
- HSP90
70 -
shMAPK15_#46
shMAPK15_#48
Fig. 3c
shSCR
250 -
shMAPK15
#46
shMAPK15
#48
Fig. 3e
shSCR
- GLI1
130 -
SAG
- + - + - +
95 -
- SMO
250 -
70 -
- GLI1
130 -
55 -
95 -
- HSP90
95 -
70 -
- HSP90
70 -
55 -
shMAPK15_#46
shMAPK15_#48
Fig. 3d
shSCR
shMAPK15
#46
shMAPK15
#48
Fig. 3f
shSCR
250 -
- + - + - +
SAG
- GLI1
130 -
250 -
95 -
- SMO
- GLI1
70 -
130 -
55 -
95 -
- HSP90
95 -
70 -
- HSP90
70 -
55 -
Figure S6. Original Western Blot images.
7

## Slide 8
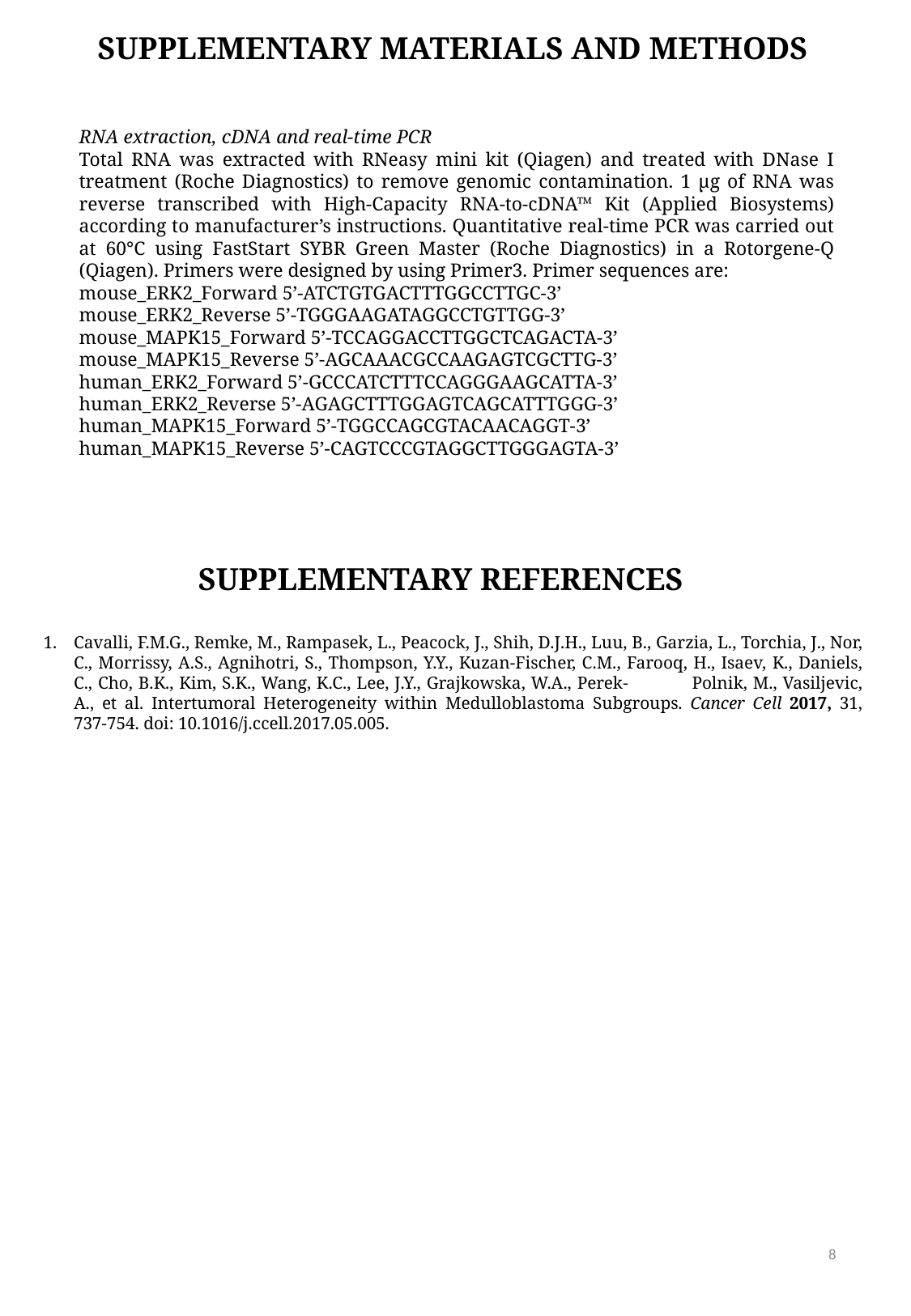

SUPPLEMENTARY MATERIALS AND METHODS
RNA extraction, cDNA and real-time PCR
Total RNA was extracted with RNeasy mini kit (Qiagen) and treated with DNase I treatment (Roche Diagnostics) to remove genomic contamination. 1 μg of RNA was reverse transcribed with High-Capacity RNA-to-cDNA™ Kit (Applied Biosystems) according to manufacturer’s instructions. Quantitative real-time PCR was carried out at 60°C using FastStart SYBR Green Master (Roche Diagnostics) in a Rotorgene-Q (Qiagen). Primers were designed by using Primer3. Primer sequences are:
mouse_ERK2_Forward 5’-ATCTGTGACTTTGGCCTTGC-3’
mouse_ERK2_Reverse 5’-TGGGAAGATAGGCCTGTTGG-3’
mouse_MAPK15_Forward 5’-TCCAGGACCTTGGCTCAGACTA-3’
mouse_MAPK15_Reverse 5’-AGCAAACGCCAAGAGTCGCTTG-3’
human_ERK2_Forward 5’-GCCCATCTTTCCAGGGAAGCATTA-3’
human_ERK2_Reverse 5’-AGAGCTTTGGAGTCAGCATTTGGG-3’
human_MAPK15_Forward 5’-TGGCCAGCGTACAACAGGT-3’
human_MAPK15_Reverse 5’-CAGTCCCGTAGGCTTGGGAGTA-3’
SUPPLEMENTARY REFERENCES
Cavalli, F.M.G., Remke, M., Rampasek, L., Peacock, J., Shih, D.J.H., Luu, B., Garzia, L., Torchia, J., Nor, C., Morrissy, A.S., Agnihotri, S., Thompson, Y.Y., Kuzan-Fischer, C.M., Farooq, H., Isaev, K., Daniels, C., Cho, B.K., Kim, S.K., Wang, K.C., Lee, J.Y., Grajkowska, W.A., Perek-	Polnik, M., Vasiljevic, A., et al. Intertumoral Heterogeneity within Medulloblastoma Subgroups. Cancer Cell 2017, 31, 737-754. doi: 10.1016/j.ccell.2017.05.005.
8
